# Analysis of adverse drug reactions in 507 cases of Tislelizumab: A real-world retrospective study based on data from Guangxi, China

**DOI:** 10.1371/journal.pone.0329464

**Published:** 2025-08-14

**Authors:** Shaohuan Lu, Dajian Chen, Yang Li, Qianxi Chen, Guangyi Meng

**Affiliations:** 1 Department of Pharmacy, The First People’s Hospital of Yulin, Yulin, Guangxi, China; 2 Adverse Drug Reaction Monitoring Office, Yulin Food and Drug Inspection and Testing Center, Yulin, Guangxi, China; Osaka University of Pharmaceutical Sciences, JAPAN

## Abstract

**Objective:**

To analyze the real-world characteristics and patterns of adverse drug reactions (ADRs) associated with tislelizumab, providing valuable insights for clinical practice.

**Methods:**

We conducted a comprehensive analysis of tislelizumab-related ADR reports within the pharmacovigilance system of Guangxi, China, spanning from 01/04/2021–31/08/2024. Our analysis focused on population characteristics, temporal distribution of ADR occurrences, system organ classes (SOCs) of serious adverse drug reactions (SADRs), profiles of major SOCs, and factors influencing SADRs and blood and lymphatic system disorders (BLSDs).

**Results:**

This study analyzed 507 tislelizumab ADR reports (698 events), including 282 SADRs (356 events), with no deaths reported. Pharmacists were the primary reporters (60.55% of reports). Most patients were aged 46–75 years (77.32%), male (72.58%), and of Han ethnicity (75.54%), and 1.78% (9/507) were of Zhuang ethnicity. A total of 86.19% of ADRs occurred within 30 days of medication. Among the SADRs, there were 83 PTs and 17 SOCs, with the most common SOCs being blood and lymphatic system disorders (15.47%, 108/698), investigations (14.90%, 104/698), hepatobiliary disorders (4.15%, 29/698), and skin and subcutaneous tissue disorders (3.15%, 22/698). Logistic regression analysis showed that chemotherapy was a significant risk factor for SADRs (OR = 4.634, 95%CI: 2.871–7.917, P < 0.001). The risk of BLSDs - related ADRs was 5.545 times higher in the chemotherapy-incorporating group than in the monotherapy group (95%CI: 3.423–8.701, P < 0.001).

**Conclusions:**

Close monitoring, particularly in patients receiving chemotherapy-incorporating regimens, is crucial during the first 30 days post-tislelizumab treatment to manage SADR risks. Proactive measures should be implemented if SADR occur.

## 1. Introduction

The advent of immune checkpoint inhibitors (ICIs) has revolutionized cancer therapy, instilling renewed hope in patients with advanced malignancies and improving their prognosis. These agents obstruct inhibitory pathways within the immune system, thereby augmenting the body’s antitumor response. Notably, inhibitors targeting programmed death 1 (PD-1), programmed cell death ligand 1 (PD-L1), and cytotoxic T-lymphocyte-associated protein 4 (CTLA-4) have been instrumental in managing various cancers [1,2]. Tislelizumab, a humanized IgG4 monoclonal antibody targeting the PD-1 receptor, has demonstrated significant activity in multiple solid tumors, leading to its rapid integration into clinical practice [3].

Common adverse drug reactions (ADRs) associated with most PD-1 inhibitors include rash, fatigue, diarrhea, and endocrine disorders [4,5]. According to the European Summary of Product Characteristics (SMPC) for tislelizumab, common ADRs include anemia (29.8%), fatigue (23.9%), and increased aspartate aminotransferase levels (21.3%). The most common Grade 3/4 ADRs were anemia (5.1%), pneumonia (4.4%), hyponatremia (2.9%), aspartate aminotransferase increased (2.6%), hypertension (2.3%), blood bilirubin increased (2.1%), pneumonitis (2.0%), and fatigue (2.0%); additionally, 1.1% of tislelizumab-treated patients had ADRs resulting in death. During the clinical development and early application phases of tislelizumab, multiple case reports documented potential severe ADRs, such as myocarditis, pneumonia, and multi-organ damage [6–17]. As of 2025, tislelizumab’s safety profile has been comprehensively evaluated using clinical trials [18–26], case reports [6–17], and FAERS database-driven post-marketing studies [27]. Although the safety profile of ICIs is well-established globally [28], and a recent FAERS-based study characterized general safety signals for tislelizumab [27], tislelizumab, as a novel anti-PD-1 agent, still lacks large-scale regional real-world data. Guangxi, an autonomous region of the Zhuang ethnic minority in China, has its own characteristics in terms of ethnic and cancer distribution, which may influence the safety and applicability of tislelizumab. Additionally, the increasing use of tislelizumab in combination therapies may further complicate its safety profile, warranting a thorough evaluation of ADRs. Currently, there is a lack of studies quantifying the risk increment of combination therapies based on real-world data.

In view of these considerations, the present study was undertaken to conduct a comprehensive analysis of 507 ADR reports related to tislelizumab collected from the pharmacovigilance system in Guangxi, China. The methodology comprised a descriptive analysis of patient demographics, timing of ADRs, and involved system organ classes (SOCs), profiles of major SOCs with and without combination therapies, supplemented by binary logistic regression to identify factors associated with SADRs and blood and lymphatic system disorders (BLSDs). Our objective was to elucidate the characteristics and patterns of ADRs associated with tislelizumab, thereby providing valuable insights into its rational and safe use in clinical practice and expanding the literature supporting the real-world safety of tislelizumab.

## 2. Methods

### 2.1. Data sourcey and processing

The pharmacovigilance system of Guangxi is a regional platform in the Guangxi Zhuang Autonomous Region of China. Its data mainly come from adverse drug reaction reports submitted voluntarily by pharmaceutical enterprises, medical institutions, drug distribution companies, consumers, and other individuals. This study included ADRs recorded by the pharmacovigilance system of Guangxi, China, between 01/04/2021 and 31/08/2024, in which tislelizumab was identified as the primary suspect. The data were accessed for research purposes on 02/09/2024.To ensure data integrity and consistency, we performed data cleaning and preprocessing on the initial dataset, which comprised 746 records. Duplicate entries were eliminated to ensure one record per unique code. We excluded reports with discrepancies in the timing of drug administration and those in which tislelizumab was not the primary suspected agent, and retained 507 reports in which tislelizumab was confirmed as the primary suspect in ADRs.

### 2.2. Definition of SADRs

The severity of ADRs was assessed based on the “severity” column in the target ADR dataset, which was assessed by reporters based on the Common Terminology Criteria for Adverse Events (CTCAE) standard. We defined ADRs marked as “severe” in the “severity” column as SADRs.

### 2.3. Data analysis

Using the Medical Dictionary for Regulatory Activities (MedDRA) 27.1, we performed fuzzy matching of adverse reaction names using lower-level terms (LLTs). Subsequently, these LLTs were mapped to the preferred terms (PTs) within MedDRA 27.1, thereby standardizing the terminology of adverse reactions in ADR reports and ensuring alignment with the corresponding system organ classes (SOCs).

In the statistical analysis, the selected tislelizumab ADRs were meticulously categorized according to sex, age, PTs, and SOCs of SADRs. For patients with multiple ADRs, each PT was meticulously documented separately.

After conducting a basic statistical analysis, we explored the factors influencing the SADRs. We used binary logistic regression to identify potential contributors to SADRs. Initially, we screened the reporting items to identify factors that could affect the occurrence of SADRs and performed a chi-square test on these factors. To assess the independence of risk factors, those with P < 0.05 in the univariate analyses were further evaluated using binary logistic regression models with forward selection. Additionally, based on our clinical experience, we considered some potentially relevant factors, even though they were not statistically significant in the chi-square test or univariate logistic regression analysis [29].Statistical analyses were performed using SPSS version 27.0.

### 2.4. Ethics statement

This study adhered to the tenets of the Declaration of Helsinki and was approved by the Ethics Committee of the First People’s Hospital of Yulin (YLSY-IRB-SR-2022043). As the analysis involved anonymous clinical data from the pharmacovigilance system of Guangxi, China, informed consent from the patients was not required and was waived by the Ethics Committee of the First People’s Hospital of Yulin.

## 3. Results

### 3.1. Basic situation of ADRs

**Fig 1:**
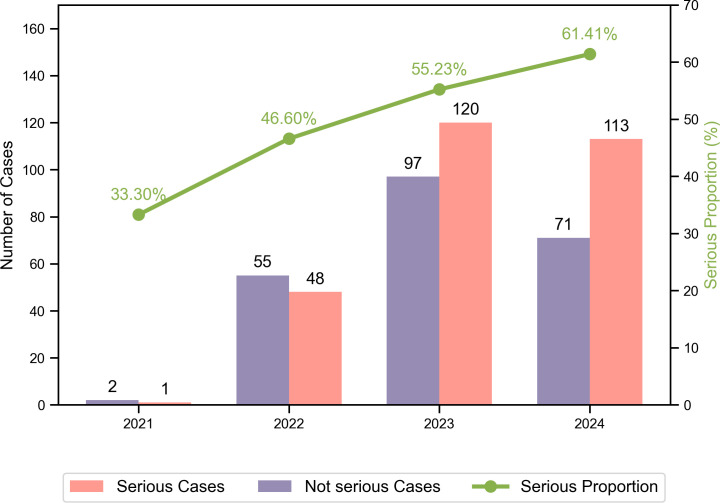
Adverse drug reaction (ADR) case distribution and serious case proportion (2021-2024). The bar chart shows the number of serious (pink) and non-serious (purple) ADR cases reported annually from 2021–2024. The green line with circular markers represents the percentage of serious cases relative to the total ADR cases each year. Numerical values above the bars indicate absolute case counts, while values above the line points show the proportion of serious cases. Data source: [S1 Table in S1 File The minimal data set of Fig 1].

**Fig 2:**
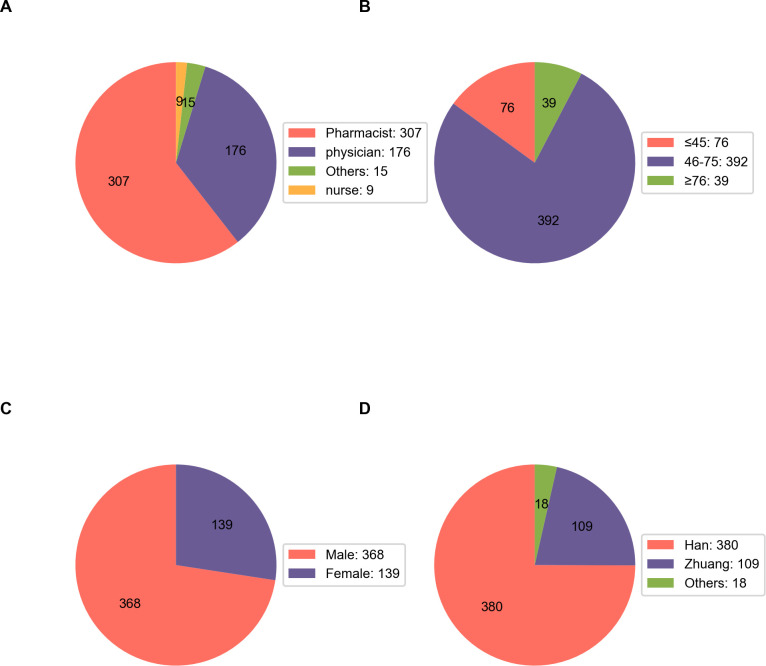
Demographic and occupational characteristics of the study population. **(A)** Occupation distribution: pharmacist (n = 307, 60.55%), physician (n = 176, 34.71%), others (n = 15, 2.96%), nurse (n = 9, 1.78%). **(B)** Age distribution: ≤ 45 years (n = 76), 46-75 years (n = 392), and ≥76 years (n = 39). **(C)** Gender distribution: male (n = 368, 72.58%), female (n = 139, 27.42%). **(D)** Ethnicity distribution: Han (n = 380, 74.95%), Zhuang (n = 109, 21.50%), others (n = 18, 3.55%). Data source: Data source: [S2 Table in S1 File The minimal data set of Fig 2].

**Fig 3:**
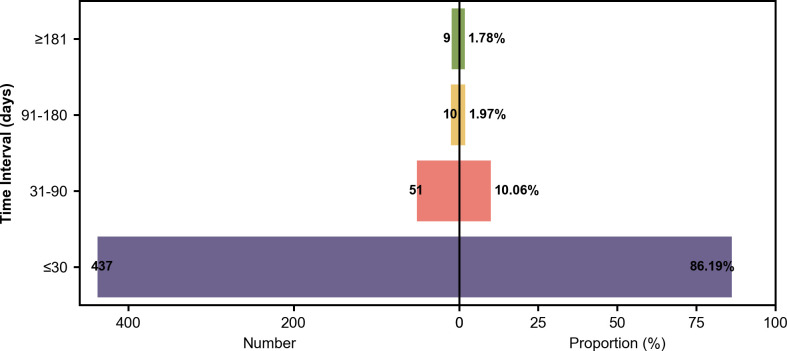
Distribution of Adverse Drug Reactions (ADRs) by Time Interval (2021-2024). Back-to-back bar charts showing the number (left) and proportion (right) of ADR cases occurring within specific time intervals after drug administration. Data sorted by the case count. Data source: [S3 Table in S1 File The minimal data set of Fig 3].

This study analyzed 507 tislelizumab ADR reports (698 events), including 282 serious ADRs (SADRs) involving 356 events. Specifically, there were 0 deaths reported among these cases. Since 2021, both the number of ADRs and the proportion of SADRs have exhibited an upward trend (Fig 1). Pharmacists emerged as the predominant reporters, accounting for 60.55% of the submissions, followed by physicians at 34.71%, and nurses at 1.78% (Fig 2A). Among the 507 reports on ADRs, Figs 2B–2D illustrate that 77.32% of patients were aged between 46 and 75 years, 72.58% were male, 75.54% were of Han nationality, and 21.50% were of Zhuang ethnicity. Regarding the temporal distribution of ADRs, 14.20% occurred on the day of medication, 86.19% occurred within a month post-medication, and only 13.81% manifested after one month (Fig 3).

### 3.2. The SOCs and PTs for ADRs

In this study, the ADRs included 20 SOCs and 122 PTs. The top 10 SOCs were as follows: investigations, blood and lymphatic system disorders, skin and subcutaneous tissue disorders, hepatobiliary disorders, gastrointestinal disorders, general disorders and administration site conditions, metabolism and nutrition disorders, cardiac disorders, immune system disorders, and respiratory, thoracic, and mediastinal disorders. Additionally, the top 30 PTs by rank were: myelosuppression, white blood cell count decreased, neutrophil count decreased, rash, pruritus, platelet count decreased, drug-induced liver injury, hypothyroidism, pyrexia, hepatic function abnormal, vomiting, nausea, anaemia, chest discomfort, autoimmune lung disease, haemoglobin decreased, neutrophils reduced, transaminases increased, chills, tachypnoea, abdominal pain, thyroid disorder, myocarditis, drug eruption, erythema, blister, asthenia, decreased appetite, liver injury, and renal impairment (S4 Table in S1 File)

### 3.3. Ranking and temporal distribution of SOCs and PTs for serious and non-serious ADRs

Specifically, there were 282 cases of serious ADRs (SADRs) (356 events) and 225 cases of non-serious ADRs (non-SADRs) (342 events). SADRs encompassed 17 SOCs and 83 PTs, whereas non-SADRs covered 18 SOCs and included 78 PTs (S4 Table in S1 File). The most prevalent SOC for SADRs was blood and lymphatic system disorders (15.47%, 108/698), followed by investigations (14.90%, 104/698), hepatobiliary disorders (4.15%, 29/698), and skin and subcutaneous tissue disorders (3.15%, 22/698). For non-SADRs, the most prominent SOC were investigations (12.61%, 88/698) and skin and subcutaneous tissue disorders (11.60%, 81/698). In terms of temporal distribution, 87.64% (321/365) of SADRs and 86.25% (294/342) of non-SADRs occurred within 30 days of drug administration. The temporal distribution of SADRs by SOCs is detailed in Table 1, and that of non-SADRs is presented in S5 Table in S1 File.

**Table 1:** Temporal Distribution of SADRs by SOCs.

SOCs	0-5 Day	11-20 Day	21-30 Day	6-10 Day	>30 Day	Total	Percentage (%)
Blood and lymphatic system disorders	12	43	25	21	7	108	15.47
Investigations	16	27	24	29	8	104	14.90
Hepatobiliary disorders	10	0	7	5	7	29	4.15
Skin and subcutaneous tissue disorders	6	5	3	4	4	22	3.15
Immune system disorders	6	3	6	0	4	19	2.72
Gastrointestinal disorders	2	3	2	4	4	15	2.15
General disorders and administration site conditions	5	6	2	0	0	13	1.86
Cardiac disorders	5	0	0	3	2	10	1.43
Metabolism and nutrition disorders	0	4	0	0	5	9	1.29
Respiratory, thoracic and mediastinal disorders	2	4	0	2	1	9	1.29
Vascular disorders	3	1	0	1	0	5	0.72
Endocrine disorders	0	1	1	1	1	4	0.57
Musculoskeletal and connective tissue disorders	2	0	1	0	0	3	0.43
Psychiatric disorders	0	2	0	0	0	2	0.29
Renal and urinary disorders	0	1	0	0	1	2	0.29
Infections and infestations	0	1	0	0	0	1	0.14
Nervous system disorders	0	1	0	0	0	1	0.14
Total	69	102	71	70	44	356	51.00

Among the top 30 PTs of SADRs, myelosuppression was the most common, comprising 14.76% (103/698) of all SADRs reported. This was followed by white blood cell count decreased (5.87%,41/698), neutrophil count decreased (2.72%,19/698), platelet count decreased (2.44%,17/698), hepatic function abnormal (1.72%,12/698), and drug-induced liver injury (1.58%,11/698). Other noteworthy manifestations included autoimmune lung disease (1.00%,7/698), myocarditis (0.72%,5/698), hypothyroidism (0.57%,4/698), and toxic epidermal necrolysis (0.29%,2/698). The top 30 PTs and their corresponding SOCs are listed in Table 2.

**Table 2:** Top 30 PTs matching SOCs of SADRs.

SOCs	PTs	Number	Percentage (%)
Blood and lymphatic system disorders	Myelosuppression	103	14.76
	White blood cell count decreased	41	5.87
	Neutrophil count decreased	19	2.72
Investigations	Platelet count decreased	17	2.44
	Hepatic function abnormal	12	1.72
Hepatobiliary disorders	Drug-induced liver injury	11	1.58
Skin and subcutaneous tissue disorders	Rash	7	1.00
Immune system disorders	Autoimmune lung disease	7	1.00
Investigations	Neutrophils reduced	7	1.00
General disorders and administration site conditions	Pyrexia	6	0.86
Investigations	Transaminases increased	6	0.86
Cardiac disorders	Myocarditis	5	0.72
Immune system disorders	Drug eruption	5	0.72
Investigations	Haemoglobin decreased	5	0.72
Gastrointestinal disorders	Abdominal pain	4	0.57
General disorders and administration site conditions	Hyperpyrexia	4	0.57
Metabolism and nutrition disorders	Hypothyroidism	4	0.57
Skin and subcutaneous tissue disorders	Pruritus	3	0.43
	Blister	3	0.43
Blood and lymphatic system disorders	Anaemia	3	0.43
Respiratory, thoracic and mediastinal disorders	Tachypnoea	3	0.43
Gastrointestinal disorders	Abdominal distension	3	0.43
Immune system disorders	Hypersensitivity	3	0.43
Vascular disorders	Anaphylactic shock	3	0.43
	Hepatitis	3	0.43
Hepatobiliary disorders	Liver injury	2	0.29
Gastrointestinal disorders	Diarrhoea	2	0.29
Renal and urinary disorders	Renal impairment	2	0.29
Cardiac disorders	Chest discomfort	2	0.29
Skin and subcutaneous tissue disorders	Toxic epidermal necrolysis	2	0.29

### 3.4. Profiles of major SOCs

#### 3.4.1. Profiles of blood and lymphatic system disorders (BLSDs).

The total number of BLSDs events was129 Among all ADRs in the tislelizumab with chemotherapy-incorporating group, the proportion of BLSDs was 31.00% (84/271), with ≥ grade 3 events at 27.68 (75/271). In the tislelizumab monotherapy group, it was 8.72% (34/390), with ≥ grade 3 events at 6.15% (24/390); refer to S6 Table in S1 File. The risk of BLSDs - related ADRs was 5.545 (95%CI: 3.423–8.701, P < 0.001) times higher in the chemotherapy-incorporating group than in the tislelizumab monotherapy group. No statistically significant differences were arose in the impact of ethnicity, tumor type, or gender on BLSDs. The analysis process is detailed in S7-9 Tables in S1 File. In the tislelizumab with chemotherapy-incorporating group, the proportions of decreased white blood cell and neutrophil counts were 60.52% (164/271) and 40.96% (111/271), respectively, with≥grade 3 events accounting for 46.13% (125/271) and 18.82% (51/271). In contrast, the tislelizumab monotherapy group exhibited decreased proportions of white blood cell and neutrophil counts of 19.49% (76/390) and 17.18% (67/390), respectively, with ≥grade 3 events at 7.44% (29/390) and 5.13% (20/390), respectively (S6 Table in S1 File). The chemotherapeutic agents frequently used in combination with tislelizumab included gemcitabine, paclitaxel, cisplatin, carboplatin, docetaxel, irinotecan, and others, refer to S10 Table in S1 File.

In our analysis, myelosuppression was the most common PT, accounting for 16.48% (115/698) of all cases, with 14.76% (103/698) reaching grade 3 or above, while anemia ranked 13 of all PTs, accounting for1.58% (11/698) of all cases, with 0.39% (2/507) reaching grade 3 above, refer to S4 Table in S1 File. Of all BLSDs and PTs related to BLSDs within the investigation SOC, only two events required corticosteroid therapy (S11 Table in S1 File).

#### 3.4.2. Profiles of hepatobiliary disorders.

In this study, 60 events (8.60%, 60/698) were documented for transaminase levels. Specifically, 53 events (7.59%, 53/698) covered aspartate aminotransferase (AST) values, with 28 events (4.01%, 28/698) reaching grade 3 or above. Additionally, 57 events (8.17%, 57/698) included ALT values, and 27 events (3.87%, 27/698) showed grade 3 or higher. Total bilirubin values were recorded in 14 events (2.01%, 14/698), with 10 events (1.43%, 10/698) presenting grade 3 or above. These cases were categorized under hepatobiliary disorders and investigations SOC categories. Under hepatobiliary disorders, the reported PTs included drug-induced liver injury (20 events), hepatic function abnormal (18 events), liver injury (4 events), hepatitis (3 events), hepatocellular injury (1 event), and jaundice (1 event). Within the investigation categories, the PTs were transaminases increased (9 events), hepatic enzyme increased (2 events), aspartate aminotransferase increased (2 events), and alanine aminotransferase increased (1 event). Among the 38 events of drug-induced liver injury and abnormal hepatic function,13 events received corticosteroid therapy. Regarding the outcomes of the 38 events of drug-induced liver injury and hepatic function abnormalities, 26 events recovered fully or improved. Refer to S11 Table in S1 File.

#### 3.4.3. Profiles of skin and subcutaneous tissue disorders.

In this study, skin and subcutaneous tissue disorders accounted for 14.76% (103/698) of all ADRs. Rash was the most common PT, reported in 4.87% (34/698) of events, with 1.00% (7/698) being serious. Toxic epidermal necrolysis, a more serious PT, was reported in 0.29% of patients (2/698). Among the 34 patients with rash events, 22 were treated with corticosteroids. All patients with blister and boxic epidermal necrolysis received corticosteroid therapy. Most of these skin disorders improved or were cured after treatment. Specific data refer in S11 Table in S1 File.

#### 3.4.3 Profiles of cardiac disorders.

Other notable ADRs included 6 events of myocarditis, which accounted for 0.86% (6/698) of all ADRs, with 4 events (0.57%, 4/698) graded as grade 3 or higher. All 6 myocarditis events were described as immune-related in the reaction narratives. Following the onset of myocarditis,3 patients discontinued tislelizumab therapy, while the treatment status of the remaining 3 remains unclear. All 6 patients received corticosteroid intervention. The outcomes were as follows: 1 was cured, 4 improved, and 1 had an unknown outcome. For more details, please refer to S11 Table in S1 File.

### 3.5. Influencing factors of SADRs

In our study of the factors influencing SADRs associated with tislelizumab, we conducted chi-square tests on variables such as age, sex, ethnicity, combination therapy, and tumor type. Ethnicity was excluded from further analysis because of its lack of statistical significance (P = 0.700) (Table 3). Despite age not showing overall significance (P = 0.071) (Table 1), it was retained in the univariate logistic regression analysis based on its clinical relevance. The analysis revealed significant effects of sex, combination therapy, and tumor type on SADRs, with age being significant in specific groups (Table 4). These variables were analyzed using a multifactorial logistic regression model with forward selectionand identified combination therapy as a risk factor for tislelizumab-associated SADRs. Specifically, the risk of SADRs was 4.634 times higher in the tislelizumab plus chemotherapy group (with or without additional therapies) than in the tislelizumab monotherapy group (OR = 4.634, P < 0.001). (Table 5). Neither ethnicity nor tumor type had a statistically significant impact on SADRs.

**Table 3:** The characteristics of patients in ADR reports for serious (SADRs) and non-serious(non-SADRs).

Category	Total (N%)	SADRs(N%)	non-SADRs(N%)	X²	P
Total	507(100%)	282	225		
Age					
≤45	80(15.8%)	52 (18.4%)	28(12.4%)	5.281	0.071
46-75	394(77.7%)	216(76.6%)	178(79.1%)		
≥76	33(6.5%)	14(5.0%)	19(8.4%)		
Gender					
Male	368(72.6%)	190 (67.4%%)	178(79.1%)	8.661	0.003
Female	139(27.4%)	92(32.6%)	47 (20.9%)		
Ethnicity					
Han	380(75.0%)	209(74.1%)	171(76.0%)	0.713	0.700
Zhuang	109(21.5%)	64(22.7%)	45(20.0%)		
Others	18(3.5%)	9(3.2%)	9(4.0%)		
Combination Therapy					
Tislelizumab monotherapy	275(54.2%)	116(41.1%)	159(70.7%)	46.204	<0.001
Tislelizumab with chemotherapy-incorporating	200(39.4%)	147(52.1%)	53(23.6%)		
Tislelizumab with others	32 (6.3%)	19 (6.7%)	13(5.8%)		
Tumor Type					
Lung cancer	116 (22.9%)	68(24.1%)	48 (21.3%)	10.894	0.12
Liver cancer	214(42.2%)	106(37.6%)	108(48.0%)		
Nasopharyngeal Carcinoma	62(12.2%)	45(16.0%)	17(7.6%)		
Others	115(22.7%)	63(22.3%)	52(23.1%)		

**Table 4:** The univariate logistic regression results for serious ADRs (SADRs).

Category	OR	95%CI	P
Age			
≤45	1.000		
46-75	0.653	0.396-1.078	0.096
≥76	0.397	0.173-0.909	0.029
Gender			
Female	1.000		
Male	0.545	0.363-0.819	0.003
Combination Therapy			
Tislelizumab monotherapy	1.000		
Tislelizumab with chemotherapy-incorporating	3.802	2.562-5.642	<0.001
Tislelizumab with others	2.003	0.951-4.220	0.068
Tumor Type			
Other Tumor	1.000		
Lung cancer	1.169	0.695-1.969	0.556
Liver cancer	0.810	0.514-1.276	0.364
Nasopharyngeal Carcinoma	2.185	1.120-4.261	0.022

**Table 5:** The multivariate logistic regression results for serious ADRs (SADRs) using forward selection in stepwise regression analysis.

	Category	OR	95%CI	P
Step 1	Combination Therapy			
	Tislelizumab monotherapy	1.000		
	Tislelizumab with chemotherapy-incorporating	3.802	2.562-5.642	<0.001
	Tislelizumab with others	2.003	0.951-4.220	0.068
Step 2	Combination Therapy			
	Tislelizumab monotherapy	1.000		
	Tislelizumab with chemotherapy-incorporating	4.585	2.981-7.053	<0.001
	Tislelizumab with others	1.843	0.861-3.949	0.116
	Tumor Type			
	Other Tumor	1.000		
	Lung cancer	2.229	1.252-3.965	0.006
	Liver cancer	0.929	0.571-1.512	0.767
	Nasopharyngeal Carcinoma	1.938	0.954-3.937	0.067
Step 3	Gender			
	Female	1.000		
	Male	0.544	0.349-0.848	0.007
	Combination Therapy			
	Tislelizumab monotherapy	1.000		
	Tislelizumab with chemotherapy-incorporating	4.634	3.001-7.156	<0.001
	Tislelizumab with others	1.871	0.868-4.032	0.110
	Tumor Type			
	Other Tumor	1.000		
	Lung cancer	2.516	1.397-4.533	0.002
	Liver cancer	1.064	0.645-1.753	0.809
	Nasopharyngeal Carcinoma	2.071	1.010-4.248	0.047

The most common form of combination therapy was with chemotherapy, which was associated with 136 SADR and 49 non-serious ADR cases (S12 Table in S1 File). The chemotherapeutic agents frequently used in combination with tislelizumab included gemcitabine, paclitaxel, cisplatin, carboplatin, docetaxel, irinotecan, and others. Targeted therapy combinations resulted in 14 SADR and 7 non-serious ADR cases, with bevacizumab being the predominant targeted agents. Combination therapies that integrated both chemotherapy and targeted therapy led to 8 SADR and 3 non-serious ADR cases. The combination of chemotherapy and radiotherapy was linked to 4 SADR and 1 non-SADR case. The detailed data on combination therapies are presented in S10 and S12 Tables in S1 File.

## 4. Discussion

This retrospective analysis examined the ADRs associated with tislelizumab in the Guangxi Zhuang Autonomous Region from 2021 to 2024. The results showed an increase in the reported ADRs, with SADRs nearly doubling to approximately 60%, particularly in patients receiving combination therapy. Specifically, the most common combination therapy was chemotherapy, which accounted for a significant proportion of SADRs. This highlights the urgent need for a comprehensive assessment of ADRs associated with this newly introduced PD-1 inhibitor, which is essential for ensuring patient safety and improving treatment efficacy.

### 4.1. Basic situation of ADRs

Our analysis found that among the 507 reports on tislelizumab-related ADRs, 77.32% of patients were aged between 46 and 75 years and 72.58% were male, which revealed a higher reporting frequency in male and older patients, particularly those aged 46–75. This is consistent with previous findings showing a protective effect in women [30,31] and may be due to a protective effect in women, reducing the risk of immune-related adverse events (irAEs) [30]. Older patients are at greater risk of ADRs; these results are consistent with the findings of Shi H [32, 33] and Pluye M[33] which reflect the increasing cancer rates associated with aging. [34]. It is important to consider age and sex when managing the risks of immunotherapy because these factors significantly influence the pharmacokinetics and severity of ADRs. Tailoring treatment strategies and increasing monitoring frequency, particularly in elderly males, are essential for optimizing the safety and efficacy of tislelizumab. This study found that 21.50% of patients were of Zhuang ethnicity (109/507), a proportion similar to the baseline population characteristics, where Zhuang ethnicity accounted for 23.6% of all inpatients in the Guangxi Zhuang Autonomous Regions.

### 4.2. Profiles of major SOC

#### 4.2.1. Blood and lymphatic system disorders (BLSDs).

In this study, the proportion of BLSDs was significantly higher in the combination chemotherapy group than in the tislelizumab monotherapy group, with 31.00% (84/271) vs. 8.72% (34/390), respectively. This finding is consistent with the official documentation of tislelizumab and other PD-1 inhibitors. Specifically, there were notable increases in the proportion of white blood cell count and neutrophil count in the tislelizumab with chemotherapy-incorporating group than in the tislelizumab monotherapy group, 60.52% 60.52% (164/271) vs. 19.49% (76/390) and 40.96% (111/271) vs. 17.18% (67/390), respectively). These findings suggest that concurrent chemotherapy markedly influences the ADR profile of tislelizumab. Our analysis of the risk factors for BLSDs also confirmed this. We found that the risk of ADRs related to BLSDs was 5.545 times higher in the chemotherapy-incorporating group than in the tislelizumab monotherapy group (OR = 5.545, 95%CI: 3.423–8.701, P < 0.001). The specific reasons for this may be that the most common form of combination therapy was chemotherapy, which included paclitaxel, docetaxel, and irinotecan. Studies have shown that chemotherapeutic agents such as paclitaxel, docetaxel, and irinotecan have well-documented hematotoxic profiles, especially in reducing hematological indicators such as white blood cell and neutrophil counts, compared to tislelizumab monotherapy [35,36]. The combination of these agents with tislelizumab may exacerbate these effects [37–39].The specific mechanism requires further research.

Discrepancies were found in the ranking of specific ADRs between this study and the official documents of tislelizumab and other PD-1 inhibitors. For example, myelosuppression was the most common grade 3/4 ADR in this study, whereas anemia was the most common grade 3/4 ADR in the SMPC document. These discrepancies may arise from variations in study designs and data collection methods between this study and the SMPC data, which can affect the ADR proportion. This study relied on voluntary reporting, which may have led to under-reporting or perspective bias. In this study, some events were reported as myelosuppression without explicitly noting anemia despite low hemoglobin levels. Clinical practice tends to document overarching conditions, such as myelosuppression, rather than specific symptoms, such as anemia.

This study shows that chemotherapy-incorporating plans are significantly linked to a higher risk of BLSD. Neither ethnicity nor tumor type had a statistically significant impact on the BLSDs. For individualized treatment, early warnings of BLSDs should focus on patients’ history of chemotherapy drug use. When tislelizumab is combined with chemotherapy, baseline blood system indicators must be recorded and monitored closely. If BLSDs occur, priority should be given to the use of granulocyte-colony-stimulating factor.

In this study, the tislelizumab monotherapy group exhibited a proportion of patients with BLSDs with ≥grade 3 events of 6.15% (24/390), but only two events of BLSDs were treated with corticosteroids. This indicates that most BLSDs were viewed as chemotherapy-related myelosuppression rather than irAEs, implying a potential under-recognition of hematological irAEs. Guidelines [40–42]from major oncology institutions suggest corticosteroids for≥grade 2 hematological irAEs. Corticosteroid treatment may be considered for patients experiencing≥grade 2 BLSDs during tislelizumab monotherapy.

#### 4.2.2. Hepatobiliary disorders.

We found that hepatobiliary disorders ranked third among SADR SOCs and fourth among overall SOCs. Most hepatobiliary disorder ADRs were reported as drug-induced liver injury and hepatic function abnormalities and primarily manifested as elevated alanine aminotransferase (ALT) and aspartate aminotransferase (AST) levels in this study. The SMPC data of tislelizumab even showed that AST increased ranks second among all ADRs. A Phase 2 clinical trial of tislelizumab indicated that hepatitis occurred in patients treated with tislelizumab and was considered an irAE [43]. Similarly, hepatobiliary disorders were consistent with the characteristics of PD-1 class drugs. Therefore, ADRs of hepatobiliary disorders are relatively expected. Based on the mechanism of PD – 1 and other ICIs, overactivation of the immune system may disrupt the normal physiological functions of the liver. Immune-related hepatitis may result from immune cell attacks on liver cells, causing elevated levels of liver enzymes. Studies have shown that ICIs can cause liver injury through multiple pathways, including the activation of cytotoxic T cells, polarization of Th cells, release of cytokines, and activation of the innate immune system [44].

Immunomodulatory therapy may be considered for some patients because of the immune-mediated nature of drug-induced liver injury [45].Among the drug-induced liver injury and hepatic function abnormal PT in this study, 34.21% (13/38) of events received corticosteroid therapy, and 68.42% (26/38) of events showed full recovery or improvement. Therefore, regular monitoring of patients’ liver function indicators, including ALT and AST levels, is crucial for promptly detecting potential liver injury and carrying out suitable interventions.

#### 4.2.3. Skin and subcutaneous tissue disorders.

This study showed that subcutaneous tissue disorders accounted for 14.76% (103/698) of all ADRs, ranking fourth among SADR SOCs and third among overall SOCs. Rash is one of the most common ADRs in skin and subcutaneous tissue disorders caused by tislelizumab in this study, reported in 4.87% (34/698) of events, with 1.00% (7/698) being serious. Toxic epidermal necrolysis, a serious PT of subcutaneous tissue disorders, was reported in 0.29% (2/698) of patients. This underscores that subcutaneous tissue disorders are characteristic ADR of tislelizumab and other PD-1 inhibitors. As a PD-1 agent, the mechanism of action of tislelizumab involves the increase in T-cell activity by blocking the PD-1 pathway, thereby regulating the immune response. Therefore, tislelizumab may exacerbate inflammatory responses in the skin and subcutaneous tissue by affecting the state of specific immune cells and activating signalling pathways [46].

Among the 34 rash events, 22 were effectively managed with corticosteroid therapy in this study. Furthermore, all patients with blisters and toxic epidermal necrolysis received corticosteroid therapy and showed improvement. These results reinforce the understanding that immune-mediated mechanisms play a significant role in these skin disorders, and the results also underscore the effectiveness of corticosteroid therapy in treating such irAE [47–49].

#### 4.2.4. Cardiac disorders.

In this study, myocarditis was identified in 6 of 698 cases (0.86%) of targeted ADRs, with 4 (0.57%) being grade 3 or higher, and all were confirmed as immune-related cases. Post-onset, all 6 patients received corticosteroid therapy, with one showing complete resolution and five demonstrating improvement. Although the proportion of myocarditis is relatively low, its severity is substantial and cannot be ignored. Although the proportion of myocarditis is low, its severity cannot be overlooked. Case reports have indicated that patients treated with tislelizumab develop severe myocarditis involving multiple organ systems [50]. These cases highlight that myocarditis is not only possible but can also lead to severe clinical outcomes such as cardiac electrical instability and heart failure [51].

### 4.3. Influencing factors of SADRs

Our results showed that combination with chemotherapy-incorporating regimens significantly increased the risk of tislelizumab-associated SADRs. The risk of SADRs was 4.634times higher in the tislelizumab plus chemotherapy group (with or without additional therapies) than in the tislelizumab monotherapy group (OR = 4.634, 95% CI: 3.001–7.156, P < 0.001). This indicates a significant increase in the risks associated with combination chemotherapy. This finding is consistent with previous studies showing that combination treatment with chemotherapy increases the risk of any grade and grade 3−5 ADRs compared to anti-PD-1/PD-L1 monotherapy [52].The mechanisms by which chemotherapy-incorporating regimens increase the risk of SADRs remain unclear. This study shows that it mainly increases SADRs by raising BLSDs, especially myelosuppression risks. This highlights the importance of a thorough review of patients’ medication history in clinical practice and the need for physicians to carefully consider the risks and benefits of different therapeutic strategies when planning treatment.

This study provides valuable insights into the real-world safety of tislelizumab; however, it has certain limitations that affect the interpretation of the results. First, the ADRs data in this study were derived from an automatic reporting system, and the total number of patients who received tislelizumab was not clear; therefore, the actual incidence of ADRs could not be accurately calculated. Second, the data from Guangxi may not be nationally representative. Third, the ADR reports in this study, mainly reported by pharmacists, may be subject to occupational perspective bias, leading to potential under-reporting of some ADRs. Despite these limitations, this study emphasizes the importance of early monitoring and caution when using tislelizumab in combination therapy, which reflects a certain degree of advancement. Future research should expand the scope of data collection, employ prospective comparative study designs, and refine the treatment strategies to enhance patient safety.

## 5. Conclusion

In conclusion, this study provides critical insights into the characteristics of all ADRs and the risk factors associatesd with tislelizumab-related SADRs and BLSDs, highlighting the importance of monitoring high-risk patients in clinical practice. The results of this study showed that most SADRs occurred within 30 days of drug administration and that there was a significant correlation between combination therapy and the reporting frequency of SADRs and BLSDs. Patients should be closely monitored for ADRs during the first 30 days after treatment with tislelizumab, with particular attention paid to patients receiving combination therapy for SADRs involving the blood, skin, and hepatobiliary tract. Proactive measures should be taken in the event of an SADR.

## Supporting information

S1 File S1 Table. The minimal data set of Fig 1. S2 Table. The minimal data set of Fig 2. S3 Table. The minimal data set of Fig 3. S4 Table. The SOCs and PTs for ADRs. S5 Table. Temporal distribution for non-serious ADRs by SOCs. S6 Table. Profiles of blood and lymphatic system disorders (BLSDs). S7 Table. The characteristics of patients in ADR reports for blood and bymphatic system disorders (BLSDs). S8 Table. The univariate logistic regression results for blood and lymphatic system disorders (BLSDs). S9 Table. The multivariate logistic regression results for blood and lymphatic system disorders using forward selection in stepwise regression analysis. S10 Table. Distribution of serious and non-serious adverse reactions in specific combination regimens of tislelizumab. S11 Table. The profiles of major SOCs. S12 Table. Tislelizumab combination program statistical data sheet. S13 Table. Summaries of official documents for PD-1 inhibitors. S14 Table. The raw data of Table 3–5 and S7-S9.
(ZIP)
