# Supplementary material for: Analysis of adverse drug reactions in 507 cases of Tislelizumab: A real-world retrospective study based on data from Guangxi, China
Source: PLoS One. 2025 Aug 14;20(8):e0329464. doi: 10.1371/journal.pone.0329464 (PMC12352749; doi:10.1371/journal.pone.0329464)
Supplement: S1 File — S1 Table. The minimal data set of Fig 1. S2 Table. The minimal data set of Fig 2. S3 Table. The minimal data set of Fig 3. S4 Table. The SOCs and PTs for ADRs. S5 Table. Temporal distribution for non-serious ADRs by SOCs. S6 Table. Profiles of blood and lymphatic system disorders (BLSDs). S7 Table. The characteristics of patients in ADR reports for blood and bymphatic system disorders (BLSDs). S8 Table. The univariate logistic regression results for blood and lymphatic system disorders (BLSDs). S9 Table. The multivariate logistic regression results for blood and lymphatic system disorders using forward selection in stepwise regression analysis. S10 Table. Distribution of serious and non-serious adverse reactions in specific combination regimens of tislelizumab. S11 Table. The profiles of major SOCs. S12 Table. Tislelizumab combination program statistical data sheet. S13 Table. Summaries of official documents for PD-1 inhibitors. S14 Table. The raw data of Table 3–5 and S7-S9. (ZIP) [file pone.0329464.s001.zip › Supporting Infomation files/S8 Table The univariate logistic regression results for Blood and Lymphatic System Disorders (BLSDs).docx]

| S8 Table The univariate logistic regression results for Blood and Lymphatic System Disorders (BLSDs) | | | |
| --- | --- | --- | --- |
| Category | OR | 95%CI | P |
| Age |  |  |  |
| ≤45 | 1.000 |  |  |
| 46-75 | 0.444 | 0.268-0.736 | 0.002 |
| ≥76 | 0.333 | 0.124-0.898 | 0.030 |
| Gender |  |  |  |
| Female | 1.000 |  |  |
| male | 0.671 | 0.435-1.035 | 0.071 |
| Combination Therapy |  |  |  |
| Tislelizumab monotherapy | 1.000 |  |  |
| Tislelizumab with chemotherapy-incorporating | 5.531 | 3.354-8.408 | <0.001 |
| Tislelizumab with others | 3.841 | 1.700-8.679 | 0.001 |
| Tumor Type |  |  |  |
| Other Tumors | 1.000 |  |  |
| Lung cancer | 0.660 | 0.366-1.191 | 0.168 |
| Liver cancer | 0.525 | 0.311-0.888 | 0.016 |
| Nasopharyngeal carcinoma | 1.763 | 0.930-3.354 | 0.082 |
